# Statistical practice and transparent reporting in the neurosciences: Preclinical motor behavioral experiments

**DOI:** 10.1371/journal.pone.0265154

**Published:** 2022-03-21

**Authors:** Olivia Hogue, Tucker Harvey, Dena Crozier, Claire Sonneborn, Abagail Postle, Hunter Block-Beach, Eashwar Somasundaram, Francis J. May, Monica Snyder Braun, Felicia L. Pasadyn, Khandi King, Casandra Johnson, Mary A. Dolansky, Nancy A. Obuchowski, Andre G. Machado, Kenneth B. Baker, Jill S. Barnholtz-Sloan

**Affiliations:** 1 Department of Quantitative Health Sciences, Lerner Research Institute, Cleveland Clinic, Cleveland, Ohio, United States of America; 2 Department of Population and Quantitative Health Sciences, School of Medicine, Case Western Reserve University, Cleveland Clinic, Cleveland, Ohio, United States of America; 3 Department of Biostatistics, School of Public Health, University of Pittsburgh, Pittsburgh, Pennsylvania, United States of America; 4 Cleveland Clinic Lerner College of Medicine, Cleveland Clinic, Cleveland, Ohio, United States of America; 5 Center for Neurological Restoration, Neurological Institute, Cleveland Clinic, Cleveland, Ohio, United States of America; 6 School of Medicine, University of Maryland, College Park, Maryland, United States of America; 7 Cleveland State University, Cleveland, Ohio, United States of America; 8 Cleveland Clinic Community Care, Cleveland, Ohio, United States of America; 9 School of Medicine, Case Western Reserve University, Cleveland, Ohio, United States of America; 10 New York-Presbyterian Hospital, Weill Cornell Medical Center, New York, New York, United States of America; 11 College of Public Health, Kent State University, Kent, Ohio, United States of America; 12 Department of Integrated Biology, Harvard College, Cambridge, Massachusetts, United States of America; 13 Frances Payne Bolton School of Nursing, Case Western Reserve University, Cleveland, Ohio, United States of America; 14 Neurological Institute, Cleveland Clinic, Cleveland, Ohio, United States of America; 15 Department of Neurosciences, Lerner Research Institute, Cleveland Clinic, Cleveland, Ohio, United States of America; 16 Center for Biomedical Informatics and Information Technology, National Cancer Institute, National Institutes of Health, Rockville, Maryland, United States of America; 17 Division of Cancer Epidemiology and Genetics, National Cancer Institute, National Institutes of Health, Rockville, Maryland, United States of America; Children’s Hospital of Los Angeles, UNITED STATES

## Abstract

Longitudinal and behavioral preclinical animal studies generate complex data, which may not be well matched to statistical approaches common in this literature. Analyses that do not adequately account for complexity may result in overly optimistic study conclusions, with consequences for reproducibility and translational decision-making. Recent work interrogating methodological shortcomings in animal research has not yet comprehensively investigated statistical shortcomings in the analysis of complex longitudinal and behavioral data. To this end, the current cross-sectional meta-research study rigorously reviewed published mouse or rat controlled experiments for motor rehabilitation in three neurologic conditions to evaluate statistical choices and reporting. Medline via PubMed was queried in February 2020 for English-language articles published January 1, 2017- December 31, 2019. Included were articles that used rat or mouse models of stroke, Parkinson’s disease, or traumatic brain injury, employed a therapeutic controlled experimental design to determine efficacy, and assessed at least one functional behavioral assessment or global evaluation of function. 241 articles from 99 journals were evaluated independently by a team of nine raters. Articles were assessed for statistical handling of non-independence, animal attrition, outliers, ordinal data, and multiplicity. Exploratory analyses evaluated whether transparency or statistical choices differed as a function of journal factors. A majority of articles failed to account for sources of non-independence in the data (74–93%) and/or did not analytically account for mid-treatment animal attrition (78%). Ordinal variables were often treated as continuous (37%), outliers were predominantly not mentioned (83%), and plots often concealed the distribution of the data (51%) Statistical choices and transparency did not differ with regards to journal rank or reporting requirements. Statistical misapplication can result in invalid experimental findings and inadequate reporting obscures errors. Clinician-scientists evaluating preclinical work for translational promise should be mindful of commonplace errors. Interventions are needed to improve statistical decision-making in preclinical behavioral neurosciences research.

## Introduction

Longitudinal and behavioral animal studies result in complex data that are often not well-matched to elementary statistical approaches. If statistical analyses do not adequately account for this complexity, study conclusions may be overly optimistic or invalid, with repercussions for translational-decision-making. Translation of novel therapies from preclinical discovery to efficacious human interventions is notoriously difficult. Though factors leading to translational failures are complex and varied, the commonplace lack of methodological rigor in preclinical animal research has been heavily researched as a contributing reason for translational failure [1–8]. Much of this research has focused on non-statistical aspects of methodological rigor (including infrequency of randomization, failure to blind assessors, and incomplete disclosure of study procedures), and on the need for greater transparency in reporting detailed methods. A recent review demonstrated that even among very high-impact publications, many lacked methodological information to inform judgment of the findings and enable replication of the experimental protocol [9], and inadequate reporting repeatedly has been found to correlate with overstated findings [10–12]. Efforts are underway to promote transparent reporting [3], increase the number of preclinical systematic reviews and meta-analyses performed [13, 14], and improve reproducibility in preclinical experiments [15].

Previous preclinical meta-research and commentary has included some attention to elements of statistical analysis and reporting [16], including disclosure of sample size calculations [4, 16], problems with small sample sizes [17–19], the effects of attrition on statistical results [20], ensuring analyses are appropriate for the experimental unit [21], calls for increased statistical education among preclinical researchers and increased collaboration with statisticians [22], and anecdotal mention of common analytical mistakes [23–25]. A recent scoping review provides likely the most complete assessment of statistical transparency to date, and found widespread problems with full statistical disclosure across the life sciences [26].

However, much of the current preclinical statistical meta-research literature still focuses on transparent reporting and how to optimize analyses using relatively straightforward study designs. A comprehensive evaluation of statistical practice for longitudinal studies specifically, and the complex data they generate, is still needed. To this end, the current study rigorously reviewed statistical decision-making and methodological reporting transparency in published preclinical mouse and rat controlled motor rehabilitation experiments for three neurologic conditions. Recommendations are provided to put results into context for practical research use.

## Methods

As this study includes only published meta-data, institutional review board approval was not required. The full dataset has been deposited in Open Science Framework at https://osf.io/dpqcu/?view_only=067709636da540329ed456382c6940af.

### Search strategy and inclusion/exclusion criteria

Medline via PubMed was queried in February 2020 for English-language articles published or available online ahead of print between January 1, 2017 and December 31, 2019. Title and Abstract and MeSH terms were included in the search. The full search syntax is provided in the Supplement. The three year time window was selected to provide a snapshot of recent practice and avoid confounds created by time.

The search strategy was initially broad to capture all articles employing rat or mouse models of any neurological disease or injury assessing improvements in motor function. Stroke, TBI, and PD were then chosen as three of the most common conditions under investigation in the returned results. Rat or mouse models were chosen to ensure relative homogeneity of sample sizes and statistical methods used in experiments, as larger animal models typically employ smaller sample sizes or single-group study design, which require different statistical methods. The search strategy did not include an explicit term for longitudinal study design, but the terms “rehabilitation” and “recovery” were expected to return articles more likely to include longitudinal data.

Included studies utilized therapeutic controlled experimental design; included at least one intervention group and one comparison group; and included at least one functional motor behavioral assessment or global evaluation of motor function. Articles with functional evaluations were chosen for two reasons. First, functional evaluations at the preclinical stage will result in outcome data more akin to the complex functional outcome data frequently used to determine efficacy in human trials. This is true in terms of clinical meaning (e.g. a test of a rodent’s ability to effectively grasp and release a small object assesses the same general construct as an assessment of a human’s ability to grasp and release a small object), as well as data structure (e.g. a preclinical behavioral task assessed multiple times over the course of treatment will generate the same type of longitudinal data as a human clinical trial with multiple data collection points). Second, limiting to one type of functional evaluation (motor) allowed article raters to be efficiently trained regarding the typical data that result from common motor evaluations. Meaning, an understanding of whether an assessment results in ordinal, count, or ratio data, whether continuous data are likely to include outliers, and so on, was necessary for raters to determine whether statistical tests may have been misapplied.

Articles were excluded if they were preventative; did not evaluate efficacy; did not include a functional outcome; were not available in English; or were retracted. One article in the dataset included the first author from this review as the study biostatistician. She did not participate in the evaluation of that article.

### Screening and data extraction

All articles were uploaded to Covidence, a systematic review software platform that enables efficient screening of article abstracts and full texts. Each title and abstract was screened independently by two of a team of three raters for the previously-detailed inclusion criteria. Using structured data collection forms, a team of nine raters then evaluated the full texts of articles. Every item was extracted independently by two different raters. Rater disagreements were settled by a third rater.

Team members with elementary formal education in statistics gathered animal details and study design information. Team members with more advanced statistical education (n = 4) evaluated statistical decision-making and transparency. Additional members of the study team extracted author and journal information. Each rater was trained by a biostatistician with expertise in analyzing complex preclinical data. Training was tailored to each rater and included education on rodent behavioral tasks and the type of data they generate and how to identify “red flags” for potential statistical insufficiencies in pre-clinical experiments (e.g. sample size inconsistencies between group allocation and presentation of results or unbalanced groups suggesting possible undisclosed attrition, unduly large error bars in figures suggesting possible outliers, etc.). A combination of assigned reading, one-on-one coaching, and group verbal instruction was used. Several practice rounds of data collection were conducted prior to beginning the study sample.

### Items evaluated

Items are derived from three best-practice reporting guidelines: the “Animal Research: Reporting of In Vivo Experiments” (ARRIVE) guidelines, endorsed by more than a thousand journals [3], the International Committee of Medical Journal Editors (ICMJE) uniform requirements for manuscripts submitted to biomedical journals [27], and the “Statistical Analyses and Methods in the Published Literature” (SAMPL) guidelines [28]. Each item is presented in the Results and Recommendations section with its rationale for inclusion in the review.

### Statistical analyses

The primary purpose of this study is descriptive and results are presented as counts with proportions. Many journals are represented in the sample multiple times, creating clustering among articles from the same journals. Thus, each article was assigned a weight, calculated by dividing the frequency of the most represented journal by the frequency of each other journal. Descriptive statistics impacted by journal clustering are reported with weighted proportions (%w) and standard error of proportion (SEP).

All inferential analyses are exploratory. When sample sizes allowed, analyses investigated whether transparent reporting differed in journals that endorse or do not endorse ARRIVE; provide or do not provide statistical reporting guidelines, require or do not require authors to disclose which authors performed which roles in the study; or are higher ranked in the journal’s discipline (per Clarivate InCites 2018 data). Discipline-specific rank was considered two ways: first, each journal was assigned the highest rank from all of its discipline-specific ranks; second, the neurosciences-specific rank was retained for the subset of journals belonging to the neurosciences.

Analyses assessing disclosure of author roles, ARRIVE endorsement, and provision of statistical reporting guidelines were conducted using Rao-Scott Chi-Square tests, a design-adjusted version of the Pearson chi-square test that accounts for non-independence arising from journal clustering and weighting [29]. Each statistical item was considered as a binary dependent variable: reported/not reported. Comparisons evaluating journal rank (continuous independent variable) were made using logistic regression with adjustments to the variance approximation to account for clustering and weighting in the data [30]. Each logistic model was evaluated for necessity of restricted cubic splines to relax the assumption of linearity (ultimately not needed as assumption was met per visual inspection of predictor by logit scatter plots).

Exploratory analyses were two-tailed with 95% confidence limits and were carried out using SAS Studio v. 3.7. Because the primary purpose of this study is to describe current practice, correction for multiple comparisons among exploratory analyses has not been made.

## Results and recommendations

Summary statistics for all figures are included in the Supplement.

### Characterization of the sample

**Fig 1:**
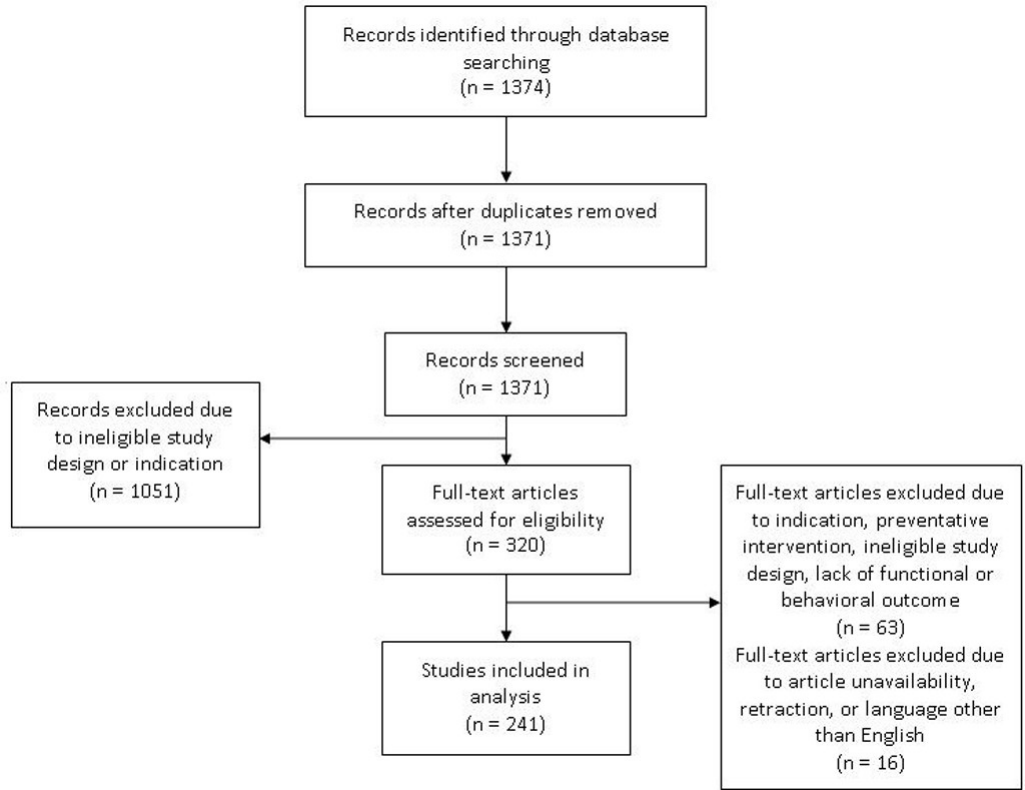
Flow diagram of articles included in the sample.

The sample included 241 articles from 99 journals (the flow diagram is presented in Fig 1). Table 1 includes a summary of basic experimental information from articles. Only thirteen percent (13%) of all items from all articles required dispute settlement by a third reviewer.

**Table 1:** Characterization of the sample: Study experimental details.

	n (%)
Animal	
Rat	149 (61.8)
Mouse	90 (37.3)
Both rat and mouse	2 (0.8)
Condition	
Stroke	140 (58.1)
Traumatic Brain Injury	56 (23.2)
Parkinson Disease	45 (18.7)
Intervention	
Drug	83 (34.4)
Biologic or stem cell	56 (23.2)
Behavioral or environmental	40 (16.6)
Neurostimulation	16 (6.6)
Supplement, vitamin, or mineral	11 (4.6)
Acupuncture, electroacupuncture, or laser acupuncture	10 (4.2)
Behavioral/environmental + drug/biologic/device	21 (8.7)
Other^a^	4 (1.7)
Functional Outcome Type^b^	
Balance and Coordination	149 (61.8)
Sensorimotor	85 (39.4)
Reaching and Forelimb	68 (28.2)
Global Neurological Rating Scales	60 (24.9)
Walking and Gait	24 (9.9)

a. n = 1 each: neurotization, low intensity focused ultrasound, low-level light emitting diode therapy (external), focal cooling over motor cortex

b. Percentages do not equal 100 because some articles included more than one functional outcome. A complete list of functional outcomes included by type can be found in the Supplement

**Fig 2:**
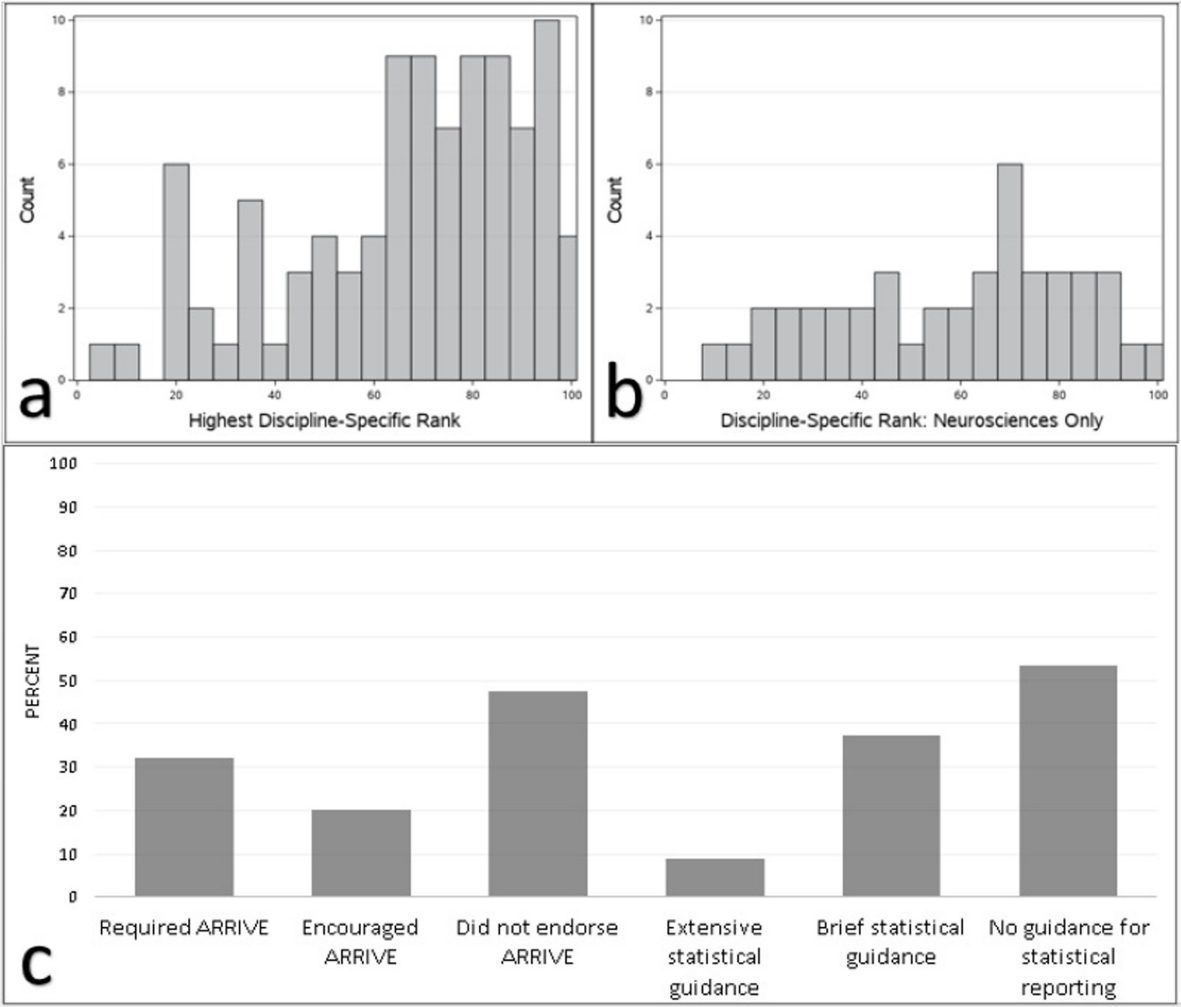
Characterization of the sample: Journal details. Proportions are unweighted and error bars are not provided, because these descriptive statistics are unaffected by article clustering within journal. **a.** Distribution of each journal’s highest discipline-specific rank (full sample, n = 241 articles). **b.** Distribution of neurosciences rank (neurosciences subsample, n = 137 articles). **c.** Journal reporting requirements (n = 99 journals).

Thirty-five journal disciplines were represented, with the highest numbers coming from neuroscience journals (137) and clinical neurology journals (48). Complete frequencies of disciplines and journals represented are in Tables S1 and S2 in S1 File. Most journals were in the top quartile for at least one discipline-specific rank (Fig 2A). The distribution of journal rank within the neurosciences was relatively uniform (Fig 2B). A majority of journals did not endorse ARRIVE guidelines and a majority did not provide guidance for reporting of statistical methods (Fig 2C). Disclosures regarding which authors performed which roles in study execution were found in 39.3%w of articles (%w indicates weighted estimate, SEP = 3.77).

### Independence: Clustering, repeated measurements, and longitudinal data

#### Rationale

Some study design factors can result in data wherein some data points are more related to one another than to others (non-independence). Independence is an assumption of most statistical tests, and transparency guidelines require confirmation that statistical assumptions are met or disclosure of analytic choices if assumptions were not met [3, 28]. Determining whether data are independent requires attention to study design and what the true experimental unit is.

Analyses for human clinical trials typically include statistical accommodations for non-independence created by study design factors such as cohort, site, and longitudinal follow-up. This is not yet commonplace in analyses of preclinical experiments in the neurosciences, where common sources of non-independence include clustering factors (such as housing multiple animals together, cohort effects, and handler effects), repeated measurements at a single time point (such as multiple behavioral trials within testing session or multiple biological samples taken per animal, the latter of which is not included in this review but detailed elsewhere), and repeated measurements over time (longitudinal data).

An experiment may contain multiple sources of non-independence. For example, a lab may run multiple sequential cohorts of animals due to space constraints, may repeat a trial of a behavioral test three times in a row per testing session, and may repeat that behavioral testing weekly for four weeks of treatment. Type I Error rate is inflated when running tests that assume independence on clustered data [31]. Averaging repeated measurements and then comparing means of means reduces power [32]. Longitudinal data analyzed without considering baseline data may be biased, and some longitudinal analysis methods have strict assumptions [33].

#### Results

**Fig 3:**
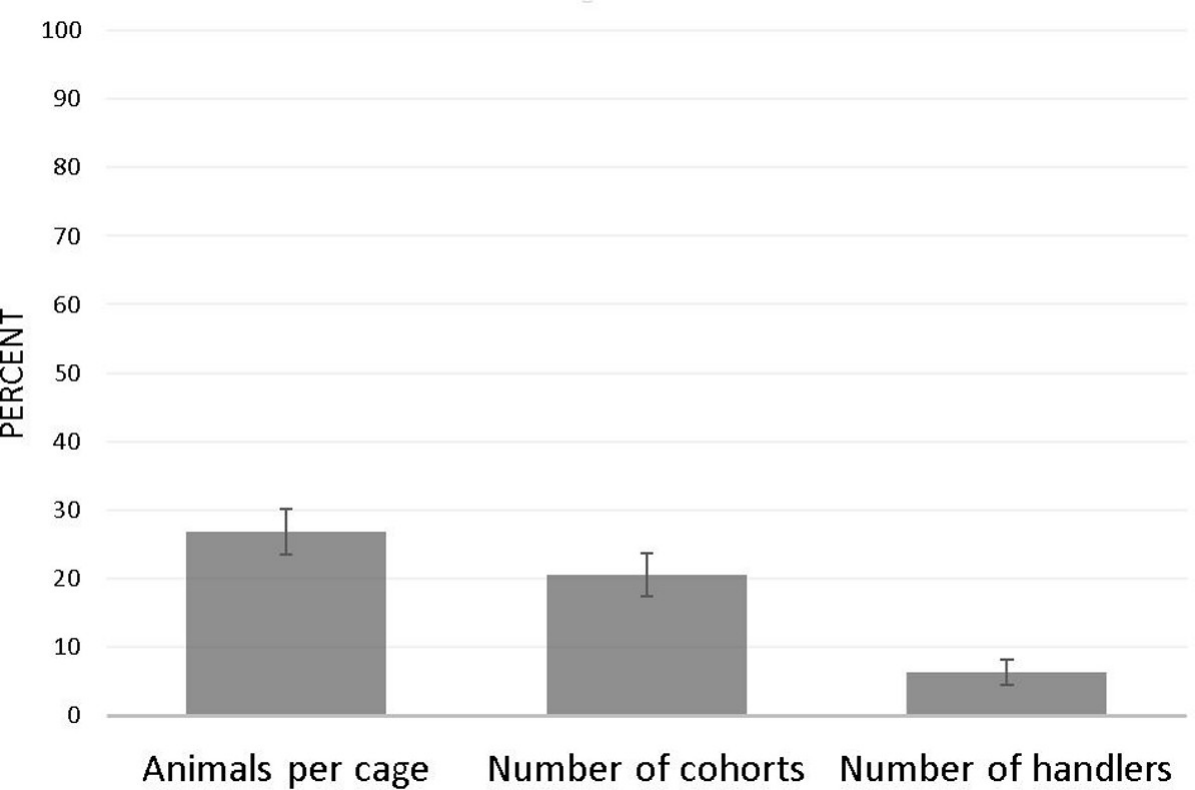
Transparent reporting of common clustering factors in animal studies (n = 241). Proportions are weighted to account for clustering of articles within journal. Error bars represent the standard error of the percentage.

**Fig 4:**
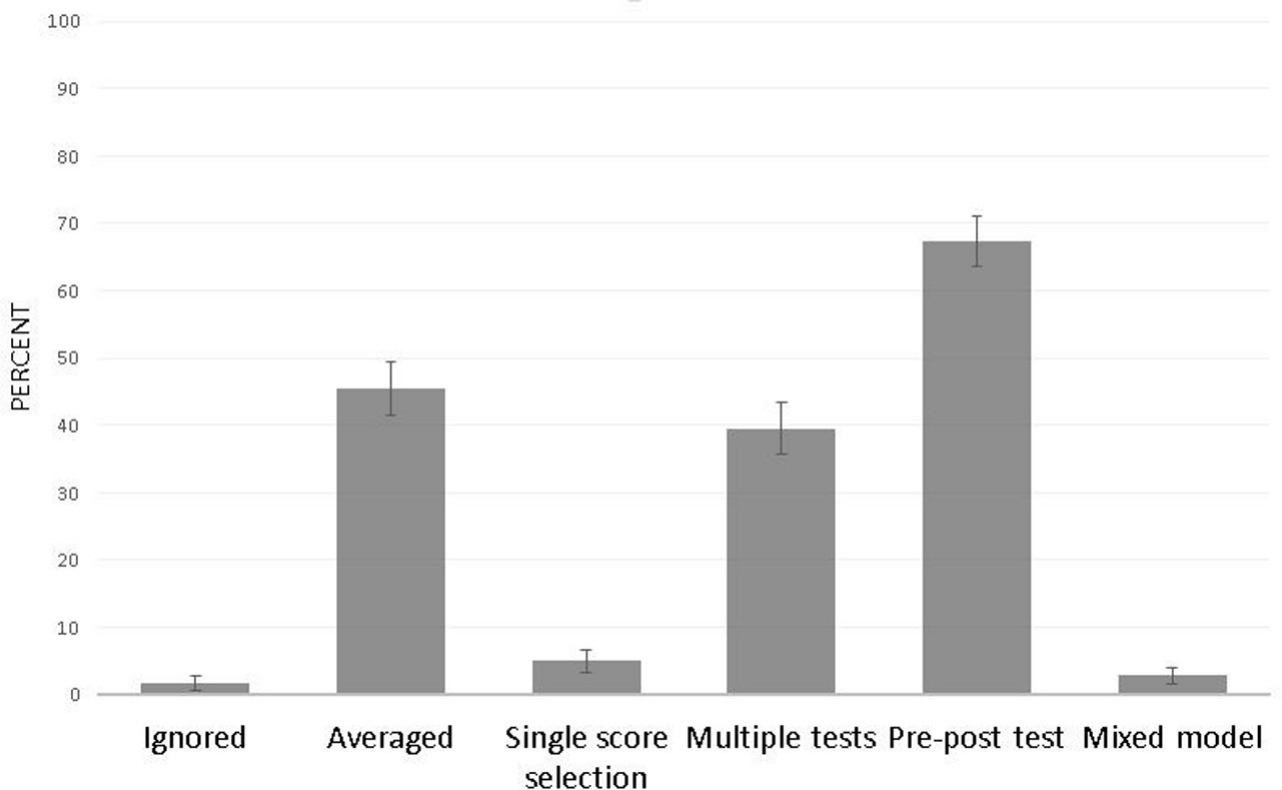
Techniques employed to account for repeated measurements (n = 227). Proportions are weighted to account for clustering of articles within journal and do not add up to 100, as multiple techniques can be used per article for different types of repeated measurements. Error bars represent the standard error of the percentage.

Report of common clustering factors was very low (Fig 3). Among the 227 articles that included repeated measurements (Fig 4), multiple behavioral trials within a single testing session were most commonly accounted for by averaging trials by session. Occasionally a single score was selected per testing session to represent each animal.

Longitudinal data was most frequently accounted for by choosing a hypothesis test that utilizes pre- and post-treatment values in the same test, such as a repeated measures analysis of variance (ANOVA). Forty percent performed multiple cross-sectional hypothesis tests (one per time point of follow-up). Of these, 4.7%w (SEP = 2.75) displayed evidence of baseline group differences, while the rest did not present any baseline data, nor confirm that groups were matched at baseline.

#### Recommendations

The low proportion of articles reporting clustering factors suggests that authors do not realize that these factors should be accounted for statistically or acknowledged as limitations, if they were not controlled in the study design. In studies with a behavioral component as were included in this review, handler effects can be especially troublesome. At minimum, these particular sources of non-independence should be disclosed and limitations acknowledged. If they are not, readers should be mindful that housing, cohort, and handler effects are possible and can result in inflated variability and imprecision of estimates.

When animals complete multiple behavioral trials within a single testing session (e.g. three wire grip tests in a row), the unit of analysis is each trial (not each animal). Trials should be nested within testing session within animal in a model that accommodates non-independence. Averaging scores from multiple trials or selecting the best score within testing session in order to satisfy the assumption of independence reduces both power and the dimensionality in the dataset and can impact results, particularly when averages are taken from a small number of trials if a high amount of trial variability exists [32].

The use of multiple cross-sectional tests for longitudinal data inflates Type I Error rate and any cross-sectional testing for longitudinal data can be subject to bias if animals are not perfectly matched at baseline. Pre-post testing is in many ways better, but carries its own limitations, including relatively strict assumptions, such as equality of variances and balanced data (discussed further in the Attrition section). Repeated measures or multivariate ANOVA also assumes equal correlations among multiple response variables, which is usually not true for longitudinal studies, where measurements taken closer to one another in time are typically more highly correlated (autoregressive structure) [34].

Depending on the study design and the resulting data structure, non-independence may be accommodated statistically using multivariate ANOVA, marginal models, or mixed effects models [35–37]. The mixed effects model, an extension of linear regression, is the most flexible and is what is frequently used in human clinical trials to accommodate multiple sources of non-independence. In cases where the assumptions of the multivariate ANOVA and the marginal model are not met and the mixed effects model is the best option, however, consultation with a statistician is recommended, as these models are easy to mis-specify.

### Attrition

#### Rationale

**Fig 5:**
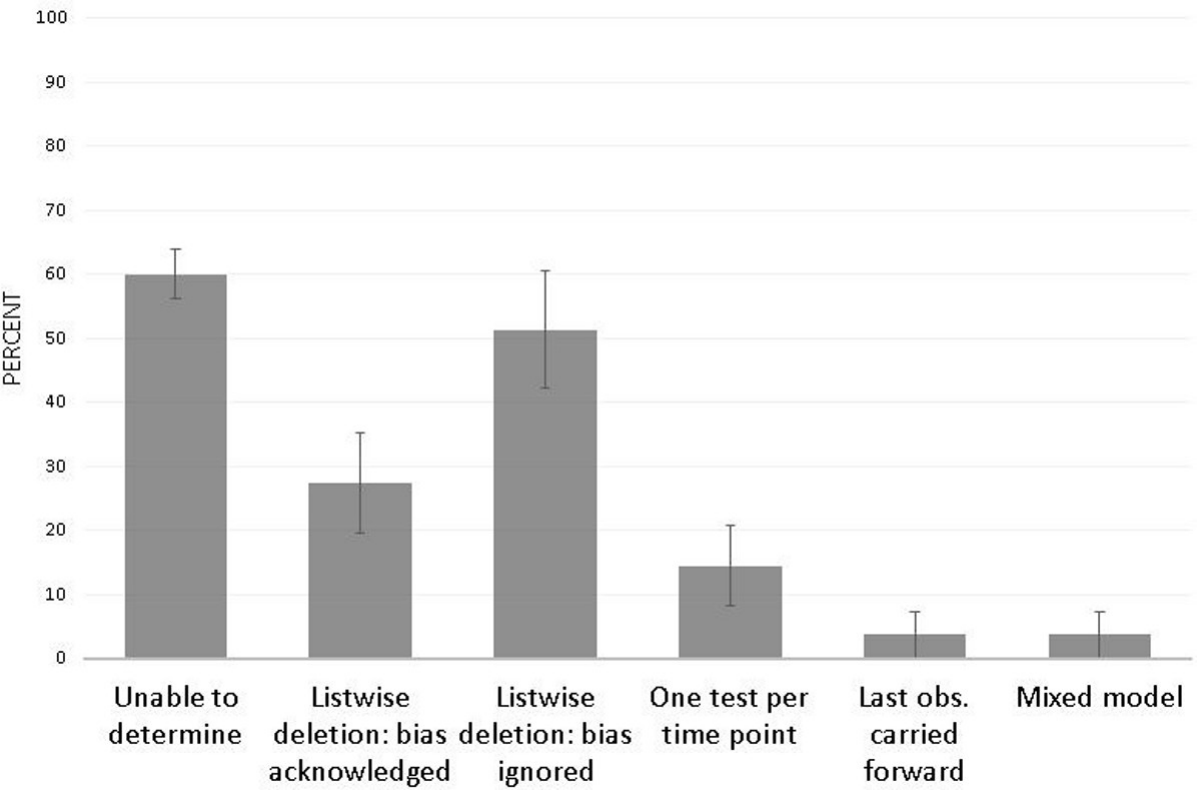
Analytic handling of animal attrition (n = 43). Listwise deletion means the analysis removes all animals without complete data, such as occurs with a repeated measures ANOVA. Proportions are weighted to account for clustering of articles within journal. Error bars represent the standard error of the percentage.

For longitudinal experiments, it is important to account for all animal deaths. Typical pre-post-treatment hypothesis tests, such as a paired t-test or repeated measures ANOVA, will not include partial data from animals that die or are removed mid-treatment. However, such treatment of these animals as “missing” does not account for the possibility that they may be poor treatment outcomes, and an analysis that could accommodate their partial data might yield different results. Attrition can inflate the chances of a false positive finding, particularly with the small sample sizes common in preclinical research [20]. *Results*. For 60%w of articles (SEP = 3.78), reviewers were unable to determine whether any animals died or were removed during the experiment. Of these, 7.4%w (SEP = 2.67) were suspected to include unreported deaths or removal of animals, because sample sizes were inconsistent between methods and results without explanation. The remainder lacked explicit statements regarding animal deaths and did not include sample sizes at both study onset and analysis. Of the 43 articles that did detail animal deaths during treatment, less than half acknowledged potential bias from data removal or attempted to include partial data in analysis, while the rest removed all data from these animals and did not acknowledge whether removal might affect results (Fig 5).

#### Recommendations

Reasons and time points for all animal attrition should be detailed. Some articles attempted to include partial animal follow-up by conducting one test per time point, including all available data for that time point. Though this does allow all data to be included, this approach inflates Type 1 error and is not recommended. Statistical methods such as mixed effects models can accommodate partial animal follow-up. These models will produce less biased estimates than tests that only include animals with complete data. For cases where partial follow-up doesn’t exist, such as when a measurement is only taken pre- and post-treatment (or only at study conclusion), it is important to report exact sample sizes at both study onset and at analysis [3, 28]. Readers should be careful to note sample sizes, particularly if sample sizes at randomization and at analysis do not match without explanation, or if group sizes are unequal without explanation.

### Continuous data: Outliers and figures

#### Rationale

ANOVA, t-tests, and linear regression are sensitive to outliers, particularly when sample sizes are small, as is frequently the case in behavioral neuroscience experiments. With small group sizes, the presence of a single animal in an intervention group with a very positive response could affect the mean response so strongly as to make the intervention appear successful as a whole if analyzed using parametric methods. Bar charts are commonly used in neuroscience publications to display continuous data. Several tutorials have been published illustrating how bar charts can obscure important aspects of the data that may support or undermine the chosen analyses, including the possible presence of outliers [38–40].

#### Results

Two hundred thirty-nine articles included an analysis assuming a continuous outcome. Of these, outliers were not mentioned in 83.2%w of articles (SEP = 2.89), and 51.3%w of articles presented continuous data using bar charts (SEP = 3.85).

#### Recommendations

Investigators should thoroughly review the nature of the data their experiments produce before analysis. Outliers should be identified and the decision-making process should be detailed regarding whether to include outlying data points and, if so, how to account for them statistically. Because bar charts do not allow adequate visualization of the data distribution, other figures should be chosen, with efforts to depict all data points whenever possible. Readers should be mindful that the results of small-sample studies analyzed using parametric methods may be impacted by undisclosed outliers.

### Ordinal data

#### Rationale

Though ordinal variables’ categories are often labeled with numbers, these are not numeric variables. The nature of an ordinal variable is such that levels do not represent equal intervals (i.e. a change from a score of 0 to 1 on an ordinal symptom scale does not have the same clinical meaning as a change from a score of 1 to 2; if it did, it would be an interval variable). Thus, ordinal variables typically should not be analyzed using a t-test or an ANOVA. Though there are occasions when ordinal variables can be treated as continuous [41], common preclinical global evaluations of neurologic function are typically not suitable candidates for this, having a small number of ordered categories and very different clinical meanings represented by the intervals between each category.

#### Results

Thirty-seven percent (36.6%w, SEP = 3.71) of the sample analyzed an ordinal variable using an analysis that requires continuous data.

#### Recommendations

An ordinal variable with just a few ordered categories can be treated as categorical. If this approach does not work for the research question, or the ordinal variable has a larger number of categories, rank-based non-parametric tests (such as Wilcoxon-Mann-Whitney or Kruskall-Wallis tests) can be used for some purposes. When a model-based approach is needed (such as for testing interactions or including covariates), a number of approaches may be used [42], including proportional odds [43], adjacent category [44], stereotype logit [45], and continuation ratio models [46]. When parametric tests are used for ordinal data, readers should be mindful that a group mean for ordinal data does not have the same clinical interpretability as a group mean for truly numeric data.

### Multiplicity

#### Rationale

Multiple hypothesis tests on the same data inflate the chances of a false positive finding. Preclinical neuroscience experiments often contain many statistical tests. It is acceptable not to correct for multiplicity for exploratory studies, but analyses should be clearly described as such and translational decision-makers should evaluate such findings with caution [47]. Confirmatory experiments should account for multiplicity.

#### Results

Zero articles applied a study-wide correction for multiple comparisons, and less than 1%w justified the reason for not doing so (0.85%w, SEP = 0.71).

#### Recommendations

It should be made clear in the statistical methods whether and how correction for multiple comparisons has been made, or the reasons for not doing so. If multiple testing is not addressed and an article contains many hypothesis tests, readers should be mindful that some “significant” results may be due to inflated Type I Error rate.

### Sample size calculations

#### Rationale

Statistical significance is difficult to put in context without understanding the size of effect for which the study was powered. Underpowered studies can result in failure to detect an effect, and if an effect is detected, it will be imprecise [17].

#### Results

Only 35 (14.7%w, SEP = 2.72) articles mentioned sample size calculation. Of these, 11 (26.5%w, SEP = 8.71) cited previously-published research as the source of data for calculations, but did not provide values used in calculations (example quote: “The sample size was chosen on the basis of our pilot experiments and those reported in previous publications” (with citation)). Six articles (17.9%w, SEP = 7.70) did not describe the source of numbers used in calculations (example quote; “Sample size in behavioral studies were assessed by power analysis using a significance level of α = 0.05 with 80% power to detect differences in ANOVA.”) Fifteen (45.7%w, SEP = 10.2) mentioned “previous data” or similar, but did not provide citation or further explanation. Four provided all values used in calculations, with three of these also including the source of values. (Example quotes are taken directly from articles in this sample.)

#### Recommendations

A transparent sample size calculation should include the size of the effect authors wish to detect (e.g. difference in means or proportions among groups), the assumed variability, desired alpha and power, and explanation regarding from where numbers were derived. Citation of previous work without including the values used in calculations is not sufficient.

### Subjective determination of statistical replicability

#### Rationale

Irreproducible statistical analyses have been proposed to be major contributors to the broader reproducibility crisis. Furthermore, statistical analyses cannot be judged for appropriateness during peer review or by readers if they are incompletely described.

#### Results

Article reviewers were asked whether they could replicate the statistical methods of each article using the details provided in the article’s methods section. Reviewers agreed on this item for 89.6% (n = 216) of articles. Only 14.5%w of articles (SEP = 2.19) were deemed by both reviewers to have reporting sufficiently complete to enable replication of statistical methods. (Due to its intentional subjective nature, this item was not subject to tie-breaking by a third reviewer.)

#### Recommendations

Statistical analyses should be described with enough detail to enable replication [3, 27, 28]. This should include all data pre-processing, methods for checking assumptions, detail of all points of decision-making, and planned interpretation of results.

### Non-statistical items

#### Rationale

This subset of non-statistical items from the ARRIVE guidelines were chosen as a reporting transparency benchmark, being low effort to report and important to disclose for reproducibility.

#### Results

**Fig 6:**
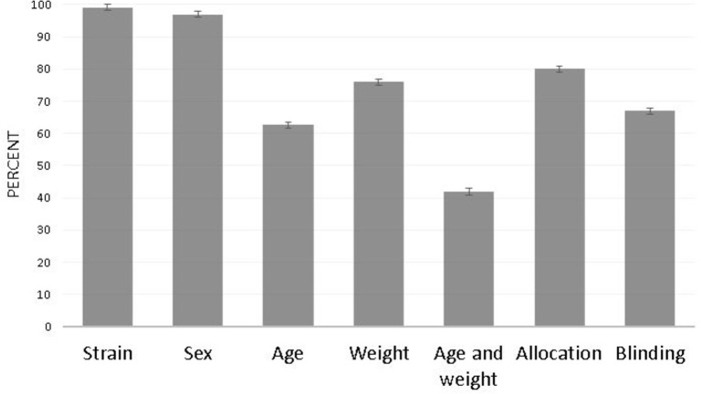
Proportions of articles in compliance with select ARRIVE guidelines, including requirement to disclose animal strain, sex, age, and weight; and allocation and blinding techniques (n = 241). Proportions are weighted to account for clustering of articles within journal. Error bars represent the standard error of the percentage.

Fig 6 displays weighted proportions of articles in compliance with each chosen ARRIVE guideline. Articles from journals not endorsing ARRIVE did not display significantly different weighted rates of reporting, relative to articles from journals requiring or recommending ARRIVE. Neither highest discipline-specific rank nor neurosciences rank was associated with ARRIVE compliance. Among the subset of articles published in journals requiring adherence to ARRIVE (n = 78), none reported all of the ARRIVE items included in this review.

#### Recommendations

The ARRIVE items evaluated in this work were selected to interrogate whether even a small group of items that should be low-effort to report are disclosed. Our findings align with previous work that found that ARRIVE compliance is low [3]. The importance of transparently reporting animal characteristics, group allocation, and blinding has been detailed extensively by others.

### Associations between statistical items and journal factors

#### Rationale

These comparisons were made to explore to what extent journal impact and reporting guidelines (endorsement of ARRIVE, provision of statistical reporting guidelines, requirement to disclose author roles) affect statistical quality and reporting.

#### Results

The majority of comparisons among each statistical item investigated and the four journal factors were non-significant, with two exceptions: 1. Articles disclosing authorship roles mentioned sample size calculations twice as often as articles that did not disclose authorship roles (69.4%w, 95% CI 50.7%-88.0%, SEP = 9.2; vs 34.1%w, 95% CI 26.3%-42.0%, SEP = 4.0). 2. Odds of mentioning sample size calculations increased 48% for every ten percentage points increase in highest rank (OR 1.48, 95% CI 1.18–1.78) and 72% for every ten percentage points increase in neurosciences rank (OR 1.72, 95% CI 1.25–2.20).

#### Recommendations

Insufficient reporting and statistical quality do not appear to be mitigated by journal reporting guidelines. More than half of journals in this sample do not provide any statistical reporting guidance and reporting was not more complete in articles from journals that do. Only reporting of sample size calculations had an association with journal requirements or rank, though mention of sample size calculations was low overall and only three articles had what would be considered completely transparent sample size justifications. Notably, though higher-ranked journals had higher odds of article mention of sample size calculations, mention of sample size calculations was not associated with journal reporting guidelines. Thus, this relationship may reflect readership norms among higher- vs lower-ranked journals, rather than journal requirements. Readers should not assume that articles from higher ranked journals or journals with more strict reporting requirements will have more complete reporting or better statistical quality.

## Discussion

Findings of the current study illustrate that in preclinical neurosciences research of interventions for three neurological conditions with a motor behavior component, statistical reporting is frequently incomplete and statistical misapplication is commonplace. Problems persist across journal disciplines and impact, and this study does not provide evidence to suggest that journal reporting requirements improve transparent reporting or statistical quality.

The statistical factors investigated in this study are not unrelated. Consider the following example: a longitudinal experiment is conducted with weekly follow-up for six weeks. In the absence of knowledge about models that can accommodate all data, the analyst must choose whether to conduct a single pre-post test, using only data from baseline and the final follow-up (thus losing all data from intermediate follow-up points, including partial data from animals that die), or to conduct multiple cross-sectional tests at each point of follow-up (thus ignoring baseline measurements and inflating Type I Error rate), or to conduct multiple pre-post tests from baseline to each point of follow-up (thus inflating Type I Error rate). Each choice carries bias that could be prevented with the use of more sophisticated statistical methods. Add in potential cage-mate, cohort, or handler effects, mishandling of ordinal data or outliers, and failure to correct for multiple testing if necessary, and the potential for invalid statistical findings increases.

### Limitations

This study was limited to motor interventions for three conditions in order to ensure relative homogeneity in the articles with regards to experimental sample sizes and design, as well as to allow training of raters in common experimental evaluation metrics so they could better evaluate statistical choices. Further, our choice of inclusion criteria resulted in a sample of articles that predominantly included complex longitudinal data. Thus, findings should be considered directly generalizable to the indications and outcomes represented; the issues discussed herein may have less relevance for other preclinical experimental designs and purposes. Additionally, our findings are based on the null hypothesis significant test (NHST) and binary interpretation of p-values predominate in preclinical work. NHST and the singular pursuit of a “significant” p-value has been widely criticized [48–50], but further discussion is outside the scope of the current work.

Article raters had varying levels of statistical expertise. Potential effects from this were mitigated by assigning raters to collect data appropriate for their levels of statistical experience, extensive and tailored training, the use of structured data collection forms, and evaluation of each data element by two or more raters. Relatedly, because statistical methods were often not completely described, article raters had to evaluate statistical choices based on only the information provided in the text. It should be made clear that results represent only the study team’s best understanding of each article’s statistical methods. Next, though we accounted for clustering of article within journal, some authors may have been included on multiple articles in the sample. This adds an additional layer of complexity to the data, which was not accounted for. Finally, journal word limits were not evaluated and this may impact level of detail in reporting. This study was not pre-registered.

## Conclusions

Statistical misapplication can result in everything from minor deviations in precision to invalid findings, and may have serious repercussions for reproducibility and translational decision-making. Clinician-scientists evaluating preclinical work for translational promise should not assume that preclinical analyses have the level of quality typical of analyses of human clinical data, nor that they received statistical review prior to publication. Interventions are needed to improve statistical decision-making in preclinical neurosciences research.

## Supporting information

S1 File (DOCX)
