# Supplementary material for: Statistical practice and transparent reporting in the neurosciences: Preclinical motor behavioral experiments
Source: PLoS One. 2022 Mar 21;17(3):e0265154. doi: 10.1371/journal.pone.0265154 (PMC8936466; doi:10.1371/journal.pone.0265154)
Supplement: S1 File — (DOCX) [file pone.0265154.s001.docx]

**Supplementary Appendix**

This document includes the following:

1. Full search terms (Figure S1)
2. Full list of rodent functional outcomes included in the sample
3. Journal disciplines (Table S1)
4. Journal titles (Table S2)
5. Summary statistics used in manuscript figures (Table S3)

Figure S1. Search syntax and number of returned results, combined Title and Abstract and MeSH search


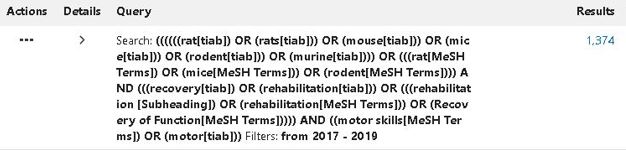


**Text reads:**

((((((rat[tiab]) OR rats[tiab])) OR (mouse[tiab])) OR (mice[tiab])) OR (rodent[tiab])) OR (murine[tiab]))) OR (((rat[MeSH Terms]) OR (mice[MeSH Terms])) OR (rodent[MeSH Terms]))) AND (((recovery[tiab]) OR (rehabilitation[tiab])) OR (((rehabilitation [Subheading]) OR (rehabilitation[MeSH Terms])) OR (Recovery of Function[MeSH Terms])))) AND ((motor skills[MeSH Terms]) OR (motor[tiab]))

Filters: from 2017 - 2019

**Extended footnote for Main Text Table 1.**

Citations included are citations provided by article authors, if applicable.

- *Balance and Coordination*: ledged tapered beam, beam walking, rotarod, inclined plane, pole test, pole climbing, tightrope test, CatWalk, computer-aided actimeter, horizontal bar test, incline test, inverted screen test, motor strength/coordination
- *Sensorimotor* : cylinder, grid walking, corner, open field, elevated body swing test, amphetamine-induced rotation, vertical grid test, adhesive removal, apomorphine-induced rotation, catalepsy test, drug-activated circling behavior
- *Reaching and Forelimb*: pellet retrieval/reaching chamber, staircase, skilled paw reaching, pasta holding, forepaw function, pasta matrix/reaching, forelimb flexion, forelimb placing, paw grasp, supination assessment task, isometric pull task, paw extension, step bias test, grip strength/wire grip
- *Global Neurological Rating Scales (as cited or described in text)*: Clark’s neurological score (Clark, 1997); Neurological severity score (Harting, 2009; Beni-Adani, 2001; Palmer, 1997; Tang 2014); Neurological scores (Wang, 2015; Maeda, 2016); ); Scoring battery described by Nakajima scoring system (Yonemi, 1998); Zea Longa Score, modified neurological severity score (Chen, 2001; Mora-Lee, 2011; Ding, 2016); Abnormal involuntary movement (AIMS) rating; Basso, Beattie, Bresnahan (BBB) score; Bederson scale; composite observational neurological score (unspecified); De Ryck’s behavioral test; Eight point performance score; Ludmila Belayey scores; multicage activity meter; neurobehavioral scores; Neurological deficit score (flexion of forelimb, flexion of hind limb, head movement 10 to the vertical axis, inability to walk straight, circling towards the paralytic side, falling to the paralytic side, and immobility) (Elvington); Neurological functional score (Garcia); 18 point scoring system neurological score (uncited)
- *Walking and Gait*: ladder rung walking, hindlimb placing, inclined plane, proprioception test, stepping test, local area network cable walking, thorax twisting, gait disturbances, Digigait, parallel rod activity chamber, gait analysis including stride length and width (Ashafaq 2012), spontaneous rotational activity, stepping test, clasping, treadmill running test

**Table S1. Journal disciplines represented**

| **Discipline** | **Count**  **(total)^a^** | **Count**  **(highest rank)^b^** |
| --- | --- | --- |
| Neurosciences | 137 | 70 |
| Clinical neurology | 48 | 27 |
| Biochemistry and molecular biology | 22 | 18 |
| Medicine, research and experimental | 22 | 11 |
| Pharmacy and pharmacology | 20 | 17 |
| Cell biology | 17 | 1 |
| Peripheral vascular disorders | 15 | 15 |
| Critical care medicine | 14 | 1 |
| Multidisciplinary sciences | 14 | 14 |
| Cell tissue engineering | 13 | 4 |
| Behavioral sciences | 12 | 11 |
| Endocrine and metabolism | 8 | 7 |
| Hematology | 8 | 2 |
| Surgery | 7 | 7 |
| Chemistry, multidisciplinary | 5 | 1 |
| Rehabilitation | 5 | 6 |
| Transplantation | 5 | 3 |
| Integrative and complementary medicine | 4 | 4 |
| Physiology | 4 | 4 |
| Biotechnology and applied microbiology | 3 | 3 |
| Chemistry, medicinal | 3 | 1 |
| Geriatrics and gerontology | 3 | 3 |
| Immunology | 3 | 0 |
| Biophysics | 2 | 1 |
| Cardiac and cardiovascular systems | 2 | 2 |
| Pathology | 2 | 2 |
| Plant sciences | 2 | 0 |
| Biology | 1 | 1 |
| Engineering, biomedical | 1 | 1 |
| Neuroimagine | 1 | 1 |
| Oncology | 1 | 0 |
| Optics | 1 | 1 |
| Sport sciences | 1 | 0 |
| Toxicology | 1 | 0 |
| Psychiatry | 1 | 1 |

1. totals do not equal 241, as many journals report multiple disciplines
2. One journal is not included in 2018 Clarivate Journal Citation Reports

**Table S2. Journals included**

| **Journal Title** | **Count** | **Percent** |
| --- | --- | --- |
| Journal of Neurotrauma | 13 | 5.39 |
| Molecular Neurobiology | 11 | 4.56 |
| Behavioural Brain Research | 10 | 4.15 |
| Journal of Stroke and Cerebrovascular Diseases | 9 | 3.73 |
| Brain Research | 8 | 3.32 |
| Scientific Reports | 7 | 2.9 |
| Experimental Neurology | 6 | 2.49 |
| Neurochemistry International | 6 | 2.49 |
| Stroke | 6 | 2.49 |
| Journal of Cerebral Blood Flow & Metabolism | 5 | 2.07 |
| Neuroscience | 5 | 2.07 |
| Neuroscience Letters | 5 | 2.07 |
| PLoS One | 5 | 2.07 |
| Translational Stroke Research | 5 | 2.07 |
| Brain Research Bulletin | 4 | 1.66 |
| International Journal of Molecular Sciences | 4 | 1.66 |
| Journal of Neuroscience | 4 | 1.66 |
| Journal of Neurosurgery | 4 | 1.66 |
| Neural Regeneration Research | 4 | 1.66 |
| Neurorehabilitation and Neural Repair | 4 | 1.66 |
| Biomedicine & Pharmacotherapy | 3 | 1.24 |
| Cell Transplantation | 3 | 1.24 |
| Frontiers in Cellular Neuroscience | 3 | 1.24 |
| Journal of Neuroinflammation | 3 | 1.24 |
| Life Sciences | 3 | 1.24 |
| Neurochemical Research | 3 | 1.24 |
| Neuropsychiatric Disease and Treatment | 3 | 1.24 |
| Neurotherapeutics | 3 | 1.24 |
| Stem Cells Translational Medicine | 3 | 1.24 |
| Aging and Disease | 2 | 0.83 |
| Brain Stimulation | 2 | 0.83 |
| CNS Neuroscience & Therapeutics | 2 | 0.83 |
| Cellular Physiology and Biochemistry | 2 | 0.83 |
| European Journal of Pharmacology | 2 | 0.83 |
| Evidence-Based Complementary and Alternative Medicine | 2 | 0.83 |
| Experimental and Therapeutic Medicine | 2 | 0.83 |
| Frontiers in Neurology | 2 | 0.83 |
| Frontiers in Neuroscience | 2 | 0.83 |
| IBRO Reports | 2 | 0.83 |
| **Journal Title** | **Count** | **Percent** |
| Journal of Cellular Physiology | 2 | 0.83 |
| Journal of Neuroscience Research | 2 | 0.83 |
| Journal of Tissue Engineering and Regenerative Medicine | 2 | 0.83 |
| Journal of the American Heart Association | 2 | 0.83 |
| Movement Disorders | 2 | 0.83 |
| Neuropharmacology | 2 | 0.83 |
| Stem Cell Research & Therapy | 2 | 0.83 |
| Stem Cells and Development | 2 | 0.83 |
| World Neurosurgery | 2 | 0.83 |
| ACS Chemical Neuroscience | 1 | 0.41 |
| Acta Neurobiologiae Experimentalis | 1 | 0.41 |
| Advances in Experimental Medicine and Biology | 1 | 0.41 |
| American Journal of Physical Medicine & Rehabilitation | 1 | 0.41 |
| Annals of Rehabilitation Medicine | 1 | 0.41 |
| BMC Neuroscience | 1 | 0.41 |
| Behavioral Neuroscience | 1 | 0.41 |
| Biochemical and Biophysical Research Communications | 1 | 0.41 |
| Brain | 1 | 0.41 |
| Brain Sciences | 1 | 0.41 |
| Brain, Behavior, and Immunity | 1 | 0.41 |
| Cell | 1 | 0.41 |
| Cells | 1 | 0.41 |
| Current Medical Science | 1 | 0.41 |
| Cytotherapy | 1 | 0.41 |
| Disease Models & Mechanisms | 1 | 0.41 |
| Endocrinology | 1 | 0.41 |
| Eneuro | 1 | 0.41 |
| Experimental Brain Research | 1 | 0.41 |
| Frontiers in Aging Neuroscience | 1 | 0.41 |
| Frontiers in Bioengineering and Biotechnology | 1 | 0.41 |
| International Journal of Molecular Medicine | 1 | 0.41 |
| International Journal of Neuroscience | 1 | 0.41 |
| Journal of Alzheimer's Disease | 1 | 0.41 |
| Journal of Biophotonics | 1 | 0.41 |
| Journal of Chemical Neuroanatomy | 1 | 0.41 |
| Journal of Huazhong University of Science and Technology | 1 | 0.41 |
| Journal of International Medical Research | 1 | 0.41 |
| Journal of Molecular Neuroscience | 1 | 0.41 |
| **Journal Title** | **Count** | **Percent** |
| Journal of Neurochemistry | 1 | 0.41 |
| Journal of Pathology | 1 | 0.41 |
| Medical Science Monitor | 1 | 0.41 |
| Metabolic Brain Disease | 1 | 0.41 |
| Molecular Therapy - Methods & Clinical Development | 1 | 0.41 |
| Molecules | 1 | 0.41 |
| NPJ Regenerative Medicine | 1 | 0.41 |
| Nature Communications | 1 | 0.41 |
| Nature Neuroscience | 1 | 0.41 |
| Neural Plasticity | 1 | 0.41 |
| NeuroImage: Clinical | 1 | 0.41 |
| NeuroToxicology | 1 | 0.41 |
| Neuroscience Bulletin | 1 | 0.41 |
| Neurosurgery | 1 | 0.41 |
| Peptides | 1 | 0.41 |
| Pharmacological Research | 1 | 0.41 |
| Phytomedicine | 1 | 0.41 |
| Planta Medica | 1 | 0.41 |
| Science Translational Medicine | 1 | 0.41 |
| Stem Cells International | 1 | 0.41 |
| Theranostics | 1 | 0.41 |
| Therapeutic Hypothermia and Temperature Management | 1 | 0.41 |

**Table S3. Summary statistics used in manuscript figures.**

| **Figure** | **Category** | **%** |  |
| --- | --- | --- | --- |
| 1c | Required ARRIVE | 32.3 |  |
|  | Encouraged ARRIVE | 20.2 |  |
|  | Did not endorse ARRIVE | 47.5 |  |
|  | Extensive statistical guidance | 9.1 |  |
|  | Brief statistical guidance | 37.4 |  |
|  | No guidance for statistical reporting | 53.5 |  |
| **Figure** | **Category** | **Weighted %** | **SEP** |
| 2a | Strain | 99.2 | 0.68 |
|  | Sex | 97 | 1.36 |
|  | Age | 62.6 | 3.72 |
|  | Weight | 76 | 3.22 |
|  | Age and weight | 41.9 | 3.8 |
|  | Allocation | 80 | 2.99 |
|  | Blinding | 67 | 3.6 |
| 2b | Animals per cage | 26.8 | 3.34 |
|  | Number of cohorts | 20.6 | 3.13 |
|  | Number of handlers | 6.3 | 1.9 |
| 2c | Ignored | 1.7 | 1.04 |
|  | R.M. averaged | 45.4 | 3.95 |
|  | R.M. single score selection | 5 | 1.73 |
|  | Multiple tests | 39.5 | 3.89 |
|  | Pre-post test | 67.3 | 3.74 |
|  | Mixed effects model | 2.8 | 1.3 |
| 3a | No mention of assumption | 83.2 | 2.93 |
|  | Mentioned but not described | 10.7 | 2.43 |
|  | Statistical test for normality | 5.3 | 1.77 |
| 3b | Incorrect | 62.6 | 4.44 |
|  | Likely incorrect | 32.1 | 4.31 |
|  | Correct | 5.3 | 2.06 |
| 3c | Incorrect | 51.1 | 4.27 |
|  | Likely incorrect | 25.5 | 3.73 |
|  | Correct | 23 | 2.62 |
| 4 | Listwise deletion: bias acknowledged | 27.3 | 7.81 |
|  | Listwise deletion: bias ignored | 51.3 | 9.19 |
|  | One test per time point | 14.5 | 6.21 |
|  | Last obs. carried forward | 3.7 | 3.63 |
|  | Mixed effects model | 3.7 | 3.63 |
